# Next-Generation Sequencing of Cerebrospinal Fluid for the Diagnosis of Neurocysticercosis

**DOI:** 10.3389/fneur.2018.00471

**Published:** 2018-06-19

**Authors:** Siyuan Fan, Xiaodong Qiao, Lei Liu, Honglong Wu, Jiali Zhou, Ruixue Sun, Qing Chen, Yan Huang, Chenhui Mao, Jing Yuan, Qiang Lu, Ying Ge, Yongjun Li, Haitao Ren, Jiawei Wang, Liying Cui, Weili Zhao, Hongzhi Guan

**Affiliations:** ^1^Department of Neurology, Peking Union Medical College Hospital, Chinese Academy of Medical Sciences and Peking Union Medical College, Beijing, China; ^2^Department of Neurology, Affiliated Hospital of Chifeng University, Chifeng, China; ^3^Department of Neurology, Beijing Tongren Hospital, Capital Medical University, Beijing, China; ^4^Tianjin Medical Laboratory, BGI-Tianjin, BGI-Shenzhen, Tianjin, China; ^5^Department of Infectious Diseases, Peking Union Medical College Hospital, Chinese Academy of Medical Sciences and Peking Union Medical College, Beijing, China; ^6^BGI Genomics, BGI-Shenzhen, Shenzhen, China

**Keywords:** neurocysticercosis, *Taenia solium*, cerebrospinal fluid, next-generation sequencing, reads

## Abstract

**Background:** Neurocysticercosis (NCC) is the most common helminthic infection of the central nervous system (CNS). The diagnosis of NCC is sometimes challenging due to its heterogenous clinical manifestations and the variable sensitivity and specificity of neuroimaging and serological tests.

**Methods:** Next-generation sequencing (NGS) of cerebrospinal fluid (CSF) was used to detect pathogens in patients with clinically suspected CNS infections. A series of patients diagnosed with NCC is reviewed here.

**Results:** Using NGS of CSF, four patients were diagnosed with NCC. The reads corresponding to *Taenia solium* ranged from 478 to 117,362, with genomic coverage of 0.0564–11.15%. Reads corresponding to *T. solium* were not found in non-template controls and far exceeded those of the background microorganisms in patients with NCC, facilitating the interpretation of the NGS results.

**Conclusions:** This case series demonstrates that NGS of CSF is promising in the diagnosis of NCC in difficult to diagnose cases. Larger studies are needed in the future.

## Introduction

Neurocysticercosis (NCC) is an infection of the central nervous system (CNS) and its meningeal coverings caused by the larval stage of the tapeworm *Taenia solium* (1). It is endemic in resource-limited areas where pigs are raised. It is the most common helminthic infection of the CNS and is a leading cause of death from food-borne diseases according to the World Health Organization (1–3). The clinical manifestations and neuroimaging findings of NCC vary greatly due to factors such as the number, stage, size, and location of parasites in the nervous system (4). The diagnostic criteria for NCC have limitations, and there is extensive discussion every time a new criterion is proposed (3, 5–7). Compared with parenchymal NCC, the diagnosis of extraparenchymal NCC is even more challenging. First, much less is known about this subtype due to its lower prevalence (3, 8). Second, lesions might not be seen on MRI because parasites in these locations have signal intensities similar to that of the cerebrospinal fluid (CSF); generally they are not enhanced by intravenous contrast, and commonly lack a scolex (9).

The use of DNA-based techniques for NCC diagnosis has been reported (4, 10, 11). A recent polymerase chain reaction (PCR) assay study showed excellent sensitivity and specificity in the diagnosis of extraparenchymal NCC (11). Next-generation sequencing (NGS) of CSF is being used increasingly for the clinical diagnosis of CNS infections (12–14). Here, we present a case series of NCC diagnosed by NGS of CSF.

## Materials and methods

### Case series

A multi-center prospective research project that includes 26 hospitals has been conducted in China since 2016, with the goal of detecting pathogens in patients with clinically suspected CNS infections using NGS of CSF. CSF from patients with encephalitis or meningitis was sent for NGS. The inclusion and exclusion criteria for encephalitis and meningitis in the multicenter study are provided in Supplementary Table 5. All of the individual-level health and medical information, including the demographic, clinical, radiological and pathogenic findings, and treatment and outcome data, were recorded in the research project database. As of December 1, 2017, 381 patients have been enrolled in the project. All of the cases reported here were derived from the database for this period.

This study was approved by the Institutional Review Board of Peking Union Medical College Hospital (PUMCH) and the Beijing Genomics Institute, Shenzhen (IRB no. JS-890). The use of the patients' clinical data and CSF samples was approved by the Ethics Committee of PUMCH. Written informed consent was obtained from each patient or their legal surrogate in accordance with the Declaration of Helsinki.

### DNA extraction

DNA was extracted from 300 μL of CSF (per patient and negative “no-template” controls) using the TIANamp Micro DNA Kit (DP316, Tiangen Biotech, Beijing, China). Proteinase K (10 mL) and buffer GB (with carrier RNA; 300 mL) were added. Then the sample was incubated for 10 min at 56°C. After adding 300 μL of cold absolute ethyl alcohol, the tube was incubated for 5 min at room temperature. The DNA-containing liquid was transferred to a new adsorption column and washed with buffer GD and buffer PW. Finally, the DNA was dissolved with 40 mL of Tris-ethylenediaminetetraacetic acid buffer.

### Library construction and sequencing

The extracted DNA was fragmented into 200–300 bp fragments using a Bioruptor Pico, according to the manufacturer's instructions. DNA libraries were constructed using through end-repair, poly(A)-tailing, adapter ligation, and PCR amplification. After quality control using an Agilent 2100 Bioanalyzer (Agilent Technologies, Santa Clara, CA, USA), the libraries were sequenced on a BGISEQ-100 platform.

### Data analysis

High-quality sequencing data were generated after removing short (<35 bp), low-quality, and low-complexity reads. The reads were then mapped to the human reference genome (hg19 and YH sequences) using the Burrows-Wheeler Aligner. The remaining data were aligned to the NCBI microbial genome database (ftp://ftp.ncbi.nlm.nih.gov/genomes/), which includes the genome sequences of 1494 bacteria, 2700 viruses, 73 fungi, and 48 parasites. The mapped data were used for further analysis. The depth and coverage were calculated for each species with Soap.coverage (SOAP).

### PCR and sanger validation

Species-specific PCR identification of *T. solium* was used to validate the NGS results. The PCR products were sequenced using an ABI PRISM 3730 DNA Sequencer (Applied Biosystems, Foster City, CA, USA). The sequences were then mapped to the NT database with the online software NCBI blast.

### *Cysticercus cellulosae* antibodies detection

The presence of *Cysticercus cellulosae* IgG in serum or the CSF sample was detected using a commercial enzyme linked immunosorbent assay (ELISA) kit using semi-purified antigen extracted from the scolices of *Taenia solium* cysticerci (Guangzhou Jianlun Biological Technology, Guangzhou, China).

## Results

### Clinical findings

Of the 381 patients with clinically suspected CNS infections, four patients with NCC were identified by NGS of CSF. Their clinical features are summarized in Table 1; and the laboratory results are summarized in Table 2. All four patients were diagnosed with probable neurocysticercosis according to the diagnostic criteria for NCC (5). Each patient's presenting medical history differed from the others.

**Table 1:** Clinical features of the four patients with neurocysticercosis.

**Case No**.	**Age range, y**.	**Delay, mo**.	**Seizure**	**Headache**	**Visual impairment**	**Transient LOC**	**Cognitive decline**	**Neuroimaging features**	**Treatment**
1	45–50	60	+	+	+	–	+	Head CT showed scattered parenchymal calcified lesions. Brain MRI showed hydrocephalus, enhancement of the basal meninges, and multiple cystic lesions in prepontine cistern and suprasellar cistern.	ABZ, DXM, ETV
2	55–60	8	–	–	+	–	–	Head CT showed a calcified lesion in the left frontal lobe. Brain MRI was normal without hydrocephalus. Spine MRI was not performed.	ABZ, DXM
3	50–55	96	–	+	+	+	+	Head CT revealed no calcified lesion. Brain MRI showed hydrocephalus and enhanced lesion posterior to the medulla.	ABZ, DXM
4	30–35	1	–	–	+	–	–	Brain MRI showed hydrocephalus and multiple cystic lesions in the suprasellar cistern.	ABZ, DXM, ETV

*Age means age at disease onset. Delay means diagnostic delay. None of these patients had focal neurological deficits. All patients came from areas where T. solium is endemic. Funduscopic examination was performed in all patients and revealed no ophthalmic parasite*.

*+, positive; –, negative; No., number; y., year; mo., month; LOC, loss of consciousness; CT, computed tomography; MRI, magnetic resonance imaging; ALB, Albendazole; DXM, dexamethasone; ETV, endoscopic third ventriculostomy*.

**Table 2:** Laboratory features of the four patients with neurocysticercosis.

**Case No**.	**CSF**							**Peripheral blood**
	**Pressure (mmH_2_O) (80–180)**	**WBC (×10^6^cells/L) (<5)**	**LYM (%) (60–70)**	**EOS (%) (1–2)**	**Protein (g/L) (0.15–0.45)**	**Glucose (mmol/L) (2.5–4.5)**	***Cysticercus cellulosae* Ab**	***Cysticercus cellulosae* Ab**
1	>330	60	60	30	0.91	1.9	+	+
2	>330	40	95	1	1.57	0.4	+	+
3	260	46	80	1	1.20	0.2	+	+
4	>330	82	70	20	0.60	2.1	+	+

*CSF results are the results before specific treatment for neurocysticercosis. All patients had negative peripheral blood human immunodeficiency virus antibodies and rapid plasma reagin. Gram staining, bacteria culture, acid-fast stain, and India-ink staining of the CSF were all negative*.

*+, positive; CSF, cerebrospinal fluid; No., number; WBC, white blood cell; LYM, lymphocyte; EOS, eosinophil; Ab, antibody*.

**Figure 1:**
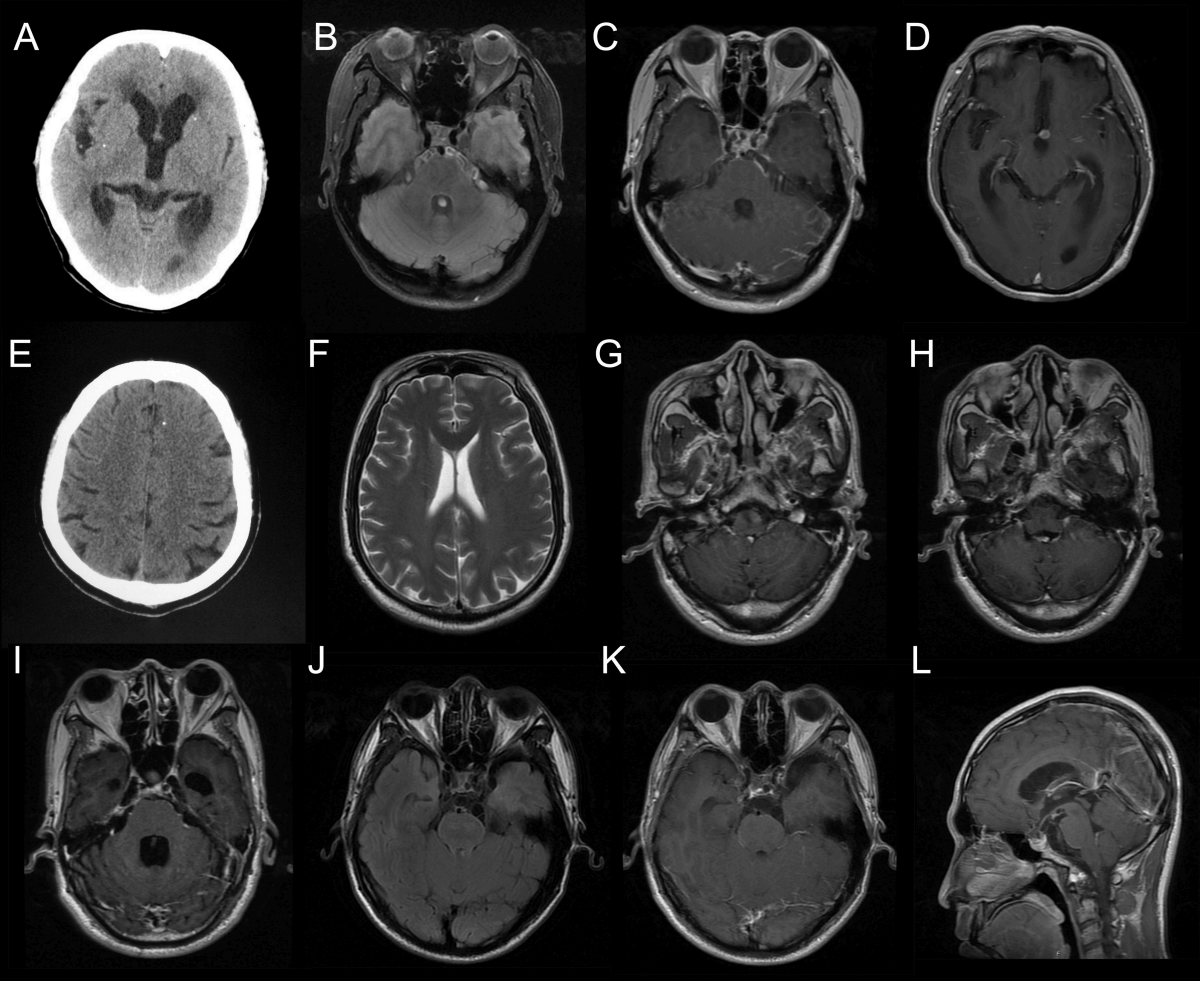
Neuroimaging results of the patients. Head computed tomography (CT) for Case 1 showed scattered parenchymal calcified lesions **(A)**. Brain magnetic resonance imaging (MRI) for Case 1 showed enhancement of the basal meninges and lesions in prepontine cistern (**B**, fluid attenuated inversion recovery, FLAIR; **C**, T1-weighted image with contrast, T1W+C) and interpeduncular cistern (**D**, T1W+C). Head CT for Case 2 showed a single calcified lesion in the left frontal lobe **(E)**. Brain MRI for Case 2 was essentially normal **(F)**. Brain MRI for Case 3 showed enhanced lesion posterior to the medulla (**G, H**, T1W+C) and hydrocephalus (**I**, T1W+C). Brain MRI for Case 4 showed multiple cystic lesions in the suprasellar cistern (**J**, FLAIR; **K,L**, T1W+C).

**Figure 2:**
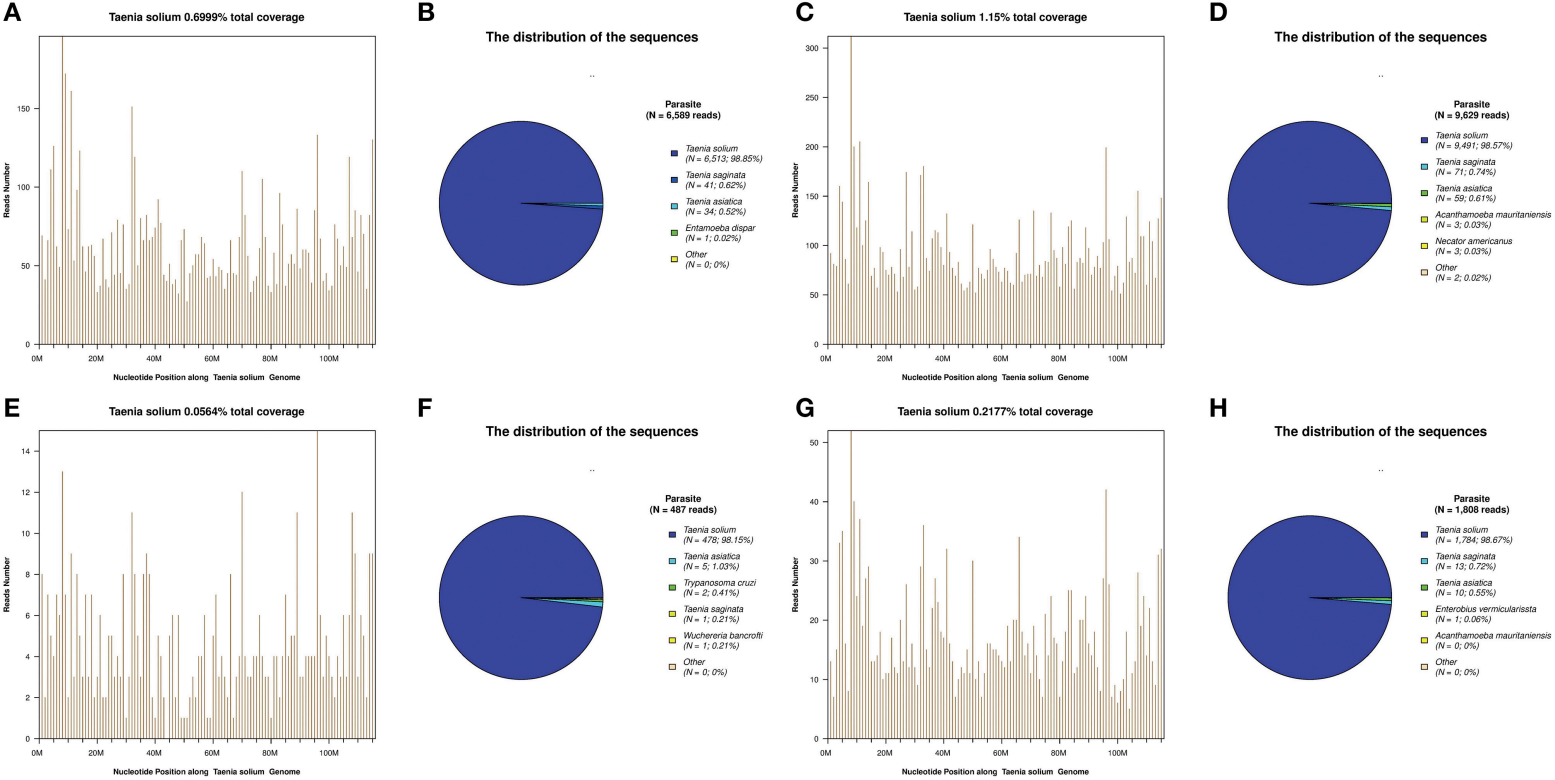
Next-generation sequencing (NGS) of cerebrospinal fluid (CSF) results for the patients. *T. solium* DNA sequences were detected in the CSF samples of all patients **(A–H)**. The identified sequence reads corresponding to *T. solium* were 6,513 **(B)**, 9,491 **(D)**, 478 **(F)**, and 1,784 **(H)**; with a genomic coverage of 0.70% **(A)**, 1.15% **(C)**, 0.06% **(E)**, and 0.22% **(G)**, for case 1, 2, 3, and 4, respectively. A few reads aligned to other members of the genus *Taenia*, including *T. saginata* and *T. asiatica* **(B,D,F,H)**.

**Case 1:** A 53-year-old woman presented with a recurrent headache, blurred vision, and progressive memory loss. The headache first appeared 5 years earlier and worsened gradually. One and a half years ago, she developed blurred vision. Brain magnetic resonance imaging (MRI) at that time showed hydrocephalus. Repeated lumbar punctures revealed increased opening pressure, elevated protein and pleocytosis without identifing the etiology. Two months ago, she developed progressive memory loss. She also had recurrent grand mal seizures about 20 years ago. On admission, head computed tomography (CT) showed scattered parenchymal calcified lesions in the right frontal lobe, right parietal lobe, right thalamus, left temporal lobe, left occipital lobe, and bilateral basal ganglia area (Figure 1A). Brain MRI showed hydrocephalus and diffuse T2-weighted hyperintensity in the juxta-ventricular white matter, together with enhancement of the meninges, especially the basal meninges, and multiple cystic lesions in the prepontine cistern, ambient cistern, and suprasellar cistern (Figures 1B–D). CSF cytology revealed increased eosinophils. NGS of CSF identified *T. solium* DNA sequences (Figures 2A,B). Therefore, the serum and CSF samples were sent for *C. cellulosae* IgG testing; and both were positive. Plain x-rays showed scattered “cigar-shaped” calcified lesions in the legs. She was diagnosed with NCC (basal subarachnoid NCC and parenchymal NCC with calcified cysts) and was treated with albendazole and dexamethasone. She also underwent an endoscopic third ventriculostomy (ETV) because of the severe hydrocephalus. The patient's symptoms, neuroimaging and CSF findings improved markedly after treatment.

**Case 2:** A 57-year-old man presented with paroxysmal blurred vision for 2 months. When he was admitted 1 month ago, lumbar puncture revealed increased opening pressure, pleocytosis, elevated protein level, and reduced glucose level. CSF cytology showed lymphocytic inflammation. Cryptococcus antigen test and Mycobacterium PCR of the CSF were negative. He was diagnosed with possible tuberculous meningitis and started on empirical anti-tuberculous treatment. However, he was readmitted after 1 month when his symptoms were not relieved. Lumbar puncture was repeated and NGS of CSF was negative. Serum and CSF samples were both positive for *C. cellulosae* IgG. Head CT revealed a single calcified lesion in the left frontal lobe (Figure 1E). Brain MRI revealed no obvious abnormalities, including hydrocephalus (Figure 1F, Supplementary Figure 2). Spine MRI was not performed. He was diagnosed with parenchymal NCC (calcified cyst), and possibly extraparenchymal NCC or spinal NCC without radiological evidence. Treatment with albendazole and dexamethasone was started. However, the symptoms and CSF findings worsened initially. To validate the diagnosis and rule out other possibilities, NGS of CSF was repeated 1.5 months later and identified *T. solium* DNA sequences (Figures 2C,D). The albendazole and dexamethasone were continued and the patient's symptoms and CSF findings improved. Note that the diagnosis of extraparenchymal NCC or spinal NCC in Case 2 was not very convincing without radiological proof. A false-positive result was not completely ruled out in this patient.

**Case 3:** A 58-year-old man presented with recurrent headache, transient loss of consciousness (LOC), and progressive memory loss. Eight years before admission, his symptoms began with recurrent headache and transient LOC. Lumbar puncture revealed increased opening pressure, pleocytosis, elevated protein level, and reduced glucose level. He was diagnosed with possible tuberculous meningitis and given empirical anti-tuberculous treatment for more than 1 year. Six years ago, he was admitted with the same symptoms and diagnosed with possible cryptococcal meningitis, for which he received fluconazole for more than 6 months and amphotericin B for 1 month. Three months before admission, he developed progressive memory loss. On admission, brain MRI showed an enhanced lesion posterior to the medulla (Figures 1G,H) and hydrocephalus (Figure 1I). NGS of CSF identified *T. solium* DNA sequence (Figures 2E,F). Plain x-rays showed scattered “cigar-shaped” calcified lesions in the legs and thoracic wall. Serum and CSF samples were both positive for *C. cellulosae* IgG antibodies. He was diagnosed with intraventricular NCC and treated with albendazole and dexamethasone. The symptoms and CSF findings subsequently improved.

**Case 4:** A 31-year-old man presented with progressive blurred vision for 3 weeks. On admission, brain MRI showed multiple cystic lesions in the suprasellar cistern (Figures 1J–L). Lumbar puncture revealed increased opening pressure, an elevated white blood cell count, elevated protein level, and reduced glucose level. CSF cytology revealed increased eosinophils. Cryptococcus antigen tests and an Xpert-MTB assay of the CSF were negative. NGS of CSF identified *T. solium* DNA sequences (Figures 2G,H). Serum and CSF were positive for *C. cellulosae* IgG antibodies. He was diagnosed with basal subarachnoid NCC and was treated with albendazole, dexamethasone, and ETV. His symptoms and CSF findings improved significantly after treatment.

### NGS of CSF result

*Taenia solium* DNA sequences were detected in the CSF samples of all four patients, but not in non-template controls (NTCs). Another 377 other patients with clinically suspected CNS infections, including patients with tuberculous meningitis, cryptococcal meningitis, and neurobrucellosis (15) were also all negative for *T. solium* (unpublished data).

For these four patients, the number of raw reads ranged from 18,777,479 to 25,216,459. The reads corresponding to *T. solium* ranged from 478 to 117,362, with genomic coverage ranging from 0.0564 to 11.15%, and the reads per million (RPM) ranging from 25.24 to 4654.18. A few reads aligned with other members of the genus *Taenia*, including *T. saginata* and *T. asiatica* (Figure 2), possibly due to the genetic similarities of these three organisms. Reads aligning with other parasites were extremely rare (Figure 2). As shown previously (15), some common background or contaminating bacteria, such as *Propionibacterium, Burkholderia*, or *Acinetobacter* are often present (Supplementary Table 2). The detailed NGS results are shown in Figure 2, Table 3, and Supplementary Tables 1–4. No viral sequences were detected in any of the four patients.

**Table 3:** The NGS of CSF of the four patients with neurocysticercosis.

**Case no**.	**Sample volume (μL)**	**DNA/RNA library**	**Raw reads**	**Species-specific reads**	**RPM**	**Genomic coverage (%)**
1	300	DNA	19,784,818	6,513	329	0.70
2	300	DNA	24,142,169	9,491	393	1.15
3	300	DNA	18,937,959	478	25.2	0.06
4	300	DNA	18,777,479	1,784	95.0	0.22

*NGS, next-generation sequencing; CSF, cerebrospinal fluid; No., number; RPM, reads per million*.

In Case 2, the initial NGS of CSF was negative for *T. solium*. Nevertheless, the diagnosis of NCC was made clinically and the patient was treated accordingly. Repeated NGS was performed when the clinical picture did not improve and showed a positive result for *T. solium* (Figures 2C,D).

The presence of *T. solium* DNA was confirmed by Sanger sequencing in Case 2 (lane 295 in Supplementary Figure 1). In the other cases, insufficient CSF was available for Sanger validation.

## Discussion

To our knowledge, this is the first case series focusing on NGS of CSF in the diagnosis of NCC.

Two major observations in our small case series are worth mentioning. First, compared with CNS bacterial infections, the NGS results for NCC are readily interpreted. As Fan et al. (15) noted, there are many background or contaminating bacteria in the results of NGS; some are present in NTCs and some are not, such as skin or body flora (15, 16). These background bacteria greatly influence the interpretation of NGS results when looking for CNS bacterial infections. In the case of NCC, however, no reads corresponding to *T. solium* were present in the NTCs, while they were abundant in our patients with NCC, greatly exceeding other possible background or contaminating microorganisms. Second, NGS results may become positive after treatment. In Case 2, NGS was negative for *T. solium* initially and the patient was still treated for NCC based on clinical judgment. The clinical features and CSF findings worsened after treatment, while the NGS results turned positive for *T. solium* when the test was repeated. The reason for the failure to detect *T. solium* DNA sequences before treatment is not clear, perhaps little DNA was released into the CSF (11, 17–19). A similar phenomenon was seen in one patient in another study evaluating DNA-based techniques; that patient had two CSF samples drawn 3 days apart, before and after treatment. The first was negative, but the second was positive (19). One likely explanation for this phenomenon is that *T. solium* DNA may have been released into the CSF when the larvae were destroyed (11, 19).

The diagnosis of extraparenchymal NCC can be challenging, not because serological tests (e.g., enzyme-linked immunoelectrotransfer blot assay) are not sufficiently accurate but, rather, because clinicians may not think to perform these tests. Note that, subarachnoid NCC presents as chronic meningitis and has the same endemic area in northern China as tuberculous meningitis. Therefore, some patients might be misdiagnosed with tuberculous meningitis and not undergo serological tests for NCC. As shown in our cases, the diagnosis of NCC was difficult in these patients with extraparenchymal NCC. The correct diagnosis was made 5 and 8 years after the onset of the initial symptoms in Cases 1 and 3, respectively. Fortunately, these patients were diagnosed with NCC “unexpectedly” with NGS of CSF, which can identify pathogens without prior information. In June 2017, a similar case demonstrating the usefulness of NGS in the diagnosis of NCC was published in *Scientific American* (20). That patient underwent a series of tests and incurred significant medical costs before the correct diagnosis was finally made with a metagenomic CSF test. The patients described here had similar experiences. For patients with atypical symptoms or neuroimaging, such as these with extraparenchymal NCC, NCC might not be suspected and serological tests might not be performed. Our case series demonstrate the superiority of NGS of CSF in these patients.

This case series is part of a multi-center research project involving some rural hospitals in China. Case 3 was referred from one of these rural hospitals. With advances in sample handling and transportation, CSF samples from less-developed areas can now reach research centers faster. NGS testing was performed in a timely manner and the result was returned to the rural hospital promptly. With the result, the hospital organized further investigations and initiated targeted treatment with good effect. To some extent, NGS of CSF may help close the gap between developed and less-developed areas in the diagnosis of difficult cases of CNS infections.

## Conclusion

This study demonstrates that NGS of CSF is promising in the diagnosis of NCC in difficult to diagnose cases. Larger studies are needed in the future.

## Author contributions

HG and SF contributed conception and design of the study. SF, XQ, LL, YH, CM, JY, QL, YG, HR, JW, LC, and WZ collected the clinical data. HW, JZ, RS, QC, and YL performed next-generation sequencing of cerebrospinal fluid and bioinformatics analysis. SF wrote the first draft of the manuscript after discussions with XQ, LL, WZ, and HG. HW, JZ, QC, and RS wrote some of the methods part. All authors contributed to manuscript revision, read and approved the submitted version.

### Conflict of interest statement

The authors declare that the research was conducted in the absence of any commercial or financial relationships that could be construed as a potential conflict of interest.

## Supplementary material

Supplementary Figure 1 PCR amplification of *T. solium* was followed by agarose gel electrophoresis to confirm *T. solium* sequences for Case 2. Lane 295 represents the result for Case 2.

Supplementary Figure 2 T2-weighted image of brain MRI for Case 2.

Supplementary Table 1 Sequencing statistics.

Supplementary Table 2 Number of reads aligning to bacterial sequences.

Supplementary Table 3 Number of reads aligning to fungal sequences.

Supplementary Table 4 Number of reads aligning to parasitic sequences.

Supplementary Table 5 Case Definitions and Exclusion Criteria for Encephalitis and Meningitis in the Multicenter Study.
