# Supplementary figures and images for: Next-Generation Sequencing of Cerebrospinal Fluid for the Diagnosis of Neurocysticercosis
Source: Front Neurol. 2018 Jun 19;9:471. doi: 10.3389/fneur.2018.00471 (PMC6018529; doi:10.3389/fneur.2018.00471)

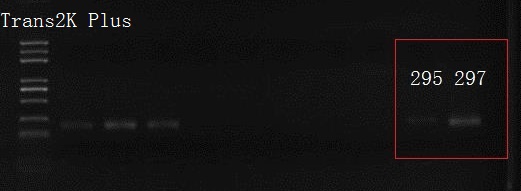

Supplement: Supplementary Figure 1 — PCR amplification of T. solium was followed by agarose gel electrophoresis to confirm T. solium sequences for Case 2. Lane 295 represents the result for Case 2. [file Image_1.JPEG]

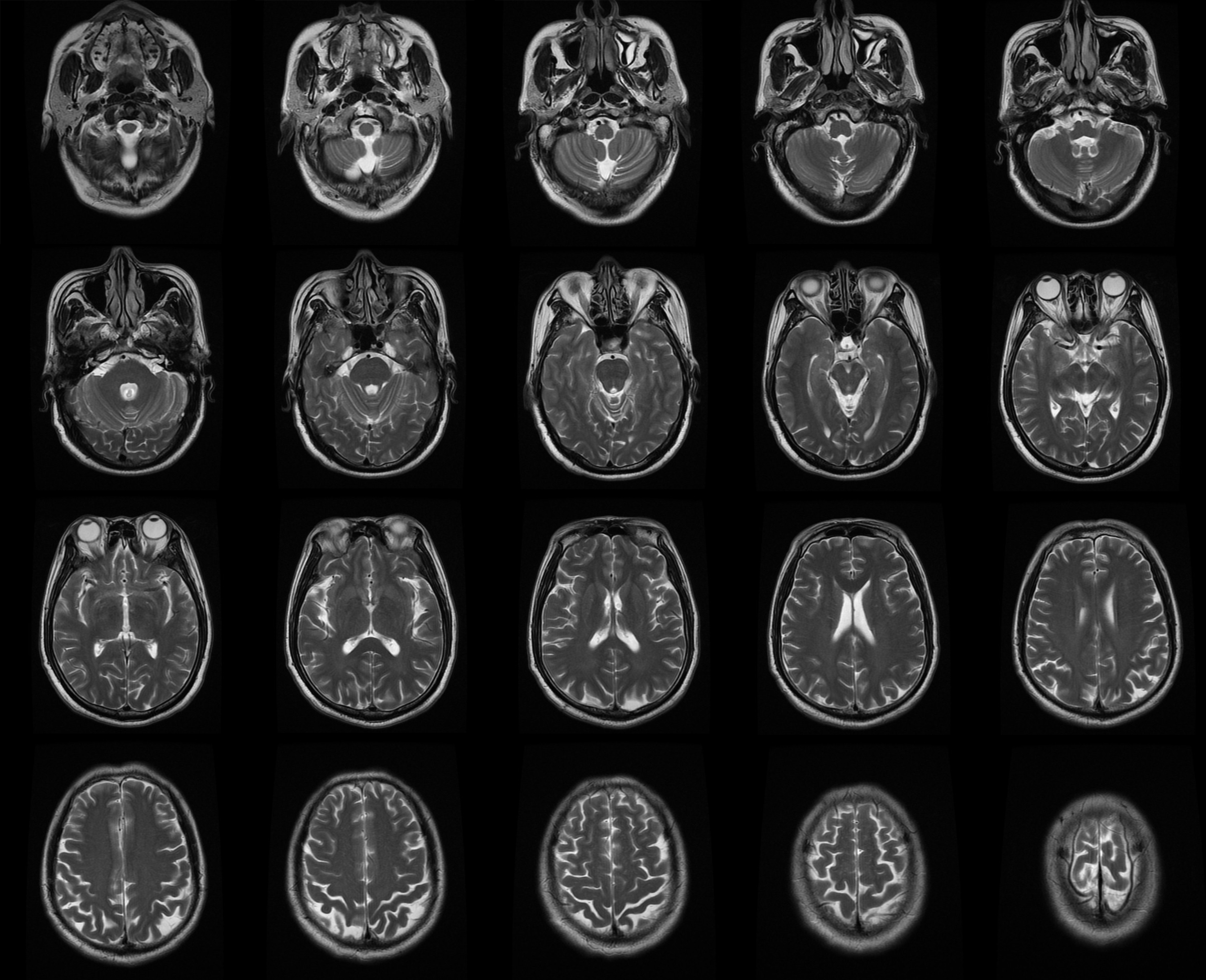

Supplement: Supplementary Figure 2 — T2-weighted image of brain MRI for Case 2. [file Image_2.JPEG]
