# Supplementary material for: Next-Generation Sequencing of Cerebrospinal Fluid for the Diagnosis of Neurocysticercosis
Source: Front Neurol. 2018 Jun 19;9:471. doi: 10.3389/fneur.2018.00471 (PMC6018529; doi:10.3389/fneur.2018.00471)
Supplement: Supplementary Table 1 — Sequencing statistics. [file Table_1.DOC]

**Supplementary Table 1. Sequencing statistics**

| **Sample**  **No.** | **Library**  **Type** | **% of Reads Passing Quality Control** | **No. of**  **Raw Reads** | **No. of Reads after**  **Quality Filtering and**  **Adapter Trimming** | **No. of Reads Aligned**  **to Human / Primate** | **No. of Non-Human /**  **Primate Reads** | **% Human /**  **Primate Reads** | **No. of Reads Aligned to**  **Microbial Genome Database** |
| --- | --- | --- | --- | --- | --- | --- | --- | --- |
| Case 1 | DNA | 97.24% | 19,784,818 | 19,238,926 | 18,965,733 | 274,111 | 98.58% | 8,703 |
| Case 2 | DNA | 98.60% | 24,142,169 | 23,803,662 | 23,388,373 | 415,289 | 98.26% | 20,206 |
| Case 3 | DNA | 98.86% | 18,937,959 | 18,721,537 | 18,525,355 | 196,182 | 98.95% | 2,839 |
| Case 4 | DNA | 98.69% | 18,777,479 | 18,531,173 | 18,296,761 | 234,412 | 98.74% | 2,870 |

No.: number; %: percentage; DNA: deoxyribonucleic acid.
