# Supplementary material for: Next-Generation Sequencing of Cerebrospinal Fluid for the Diagnosis of Neurocysticercosis
Source: Front Neurol. 2018 Jun 19;9:471. doi: 10.3389/fneur.2018.00471 (PMC6018529; doi:10.3389/fneur.2018.00471)
Supplement: Supplementary Table 2 — Number of reads aligning to bacterial sequences. [file Table_2.DOC]

**Supplementary Table 2. Number of reads aligning to bacterial sequences.**

**Data for** Case 1

| **Species** | **Genomic Coverage** | **Aligned Reads** |
| --- | --- | --- |
| *Propionibacterium_acnes* | 6599/2560282 | 52 |
| *Propionibacterium_humerusii* | 777/2644116 | 4 |
| *Propionibacterium_propionicum* | 103/3449360 | 1 |
| *Brevundimonas_vesicularis* | 6706/3358839 | 54 |
| *Burkholderia_multivorans* | 297/7281887 | 1 |
| *Burkholderia_stabilis* | 118/8527967 | 1 |
| *Acinetobacter_johnsonii* | 1323/3509795 | 9 |
| *Acinetobacter_baumannii* | 249/4086879 | 2 |
| *Acinetobacter_junii* | 256/3782588 | 2 |
| *Acinetobacter_schindleri* | 244/3415006 | 2 |
| *Acinetobacter_parvus* | 116/3606729 | 1 |
| *Acinetobacter_radioresistens* | 119/3195606 | 1 |
| *Staphylococcus_capitis* | 473/2466594 | 4 |
| *Staphylococcus_epidermidis* | 461/2616530 | 3 |
| *Staphylococcus_equorum* | 196/2753539 | 2 |
| *Staphylococcus_hominis* | 170/1140916 | 2 |
| *Staphylococcus_massiliensis* | 248/2366665 | 2 |
| *Staphylococcus_cohnii* | 124/2677922 | 1 |
| *Staphylococcus_gallinarum* | 122/3174430 | 1 |
| *Staphylococcus_haemolyticus* | 124/2685015 | 1 |
| *Staphylococcus_saprophyticus* | 126/2516575 | 1 |
| *Streptococcus_gordonii* | 306/2196662 | 2 |
| *Streptococcus_I_P16* | 114/2023580 | 1 |
| *Streptococcus_oralis* | 357/1958690 | 1 |
| *Streptococcus_pseudopneumoniae* | 248/2190731 | 1 |
| *Streptococcus_sanguinis* | 133/2388435 | 1 |
| *Thermobifida_fusca* | 1446/3642249 | 13 |
| *Acidovorax_KKS102* | 1532/5196935 | 12 |
| *Enhydrobacter_aerosaccus* | 4350/2856138 | 12 |
| *Corynebacterium_matruchotii* | 488/2992345 | 4 |
| *Corynebacterium_propinquum* | 79/2553780 | 1 |
| *Corynebacterium_vitaeruminis* | 119/2931780 | 1 |
| *Moraxella_osloensis* | 2965/2434688 | 9 |
| *Bifidobacterium_thermophilum* | 927/2291643 | 8 |
| *Lactobacillus_crispatus* | 260/2043161 | 4 |
| *Lactobacillus_fermentum* | 267/2297851 | 2 |
| *Prevotella_loescheii* | 243/3508889 | 2 |
| *Sphingomonas_MM* | 158/4054833 | 2 |
| *Sphingomonas_adhaesiva* | 351/4026130 | 2 |
| *Sphingomonas_melonis* | 760/4156476 | 2 |
| *Lactobacillus_brevis* | 127/2395134 | 1 |
| *Lactobacillus_plantarum* | 131/3418468 | 1 |
| *Prevotella_copri* | 121/3512733 | 1 |
| *Prevotella_multisaccharivorax* | 117/3388664 | 1 |
| *Prevotella_veroralis* | 123/2994185 | 1 |
| *Sphingomonas_echinoides* | 75/4264986 | 1 |
| *Sphingomonas_parapaucimobilis* | 103/3995782 | 1 |
| *Pseudomonas_TKP* | 124/7012672 | 1 |
| *Pseudomonas_fluorescens* | 127/6136735 | 1 |
| *Pseudomonas_mendocina* | 110/5434353 | 1 |
| *Pseudomonas_poae* | 123/5512241 | 1 |
| *Pseudomonas_stutzeri* | 130/4650155 | 1 |
| *Cutibacterium_granulosum* | 376/2141325 | 3 |
| *Propionibacterium_namnetense* | 469/2369944 | 3 |
| *Neisseria_perflava* | 141/3786149 | 1 |
| *Neisseria_sicca* | 267/2831372 | 1 |
| *Cloacibacterium_normanense* | 731/2736686 | 5 |
| *Haemophilus_parahaemolyticus* | 128/2038028 | 2 |
| *Haemophilus_parainfluenzae* | 250/2086875 | 2 |
| *Enterococcus_cecorum* | 500/2438959 | 4 |
| *Ralstonia_insidiosa* | 348/5808308 | 3 |
| *Peptoniphilus_harei* | 280/1874575 | 1 |
| *Ralstonia_mannitolilytica* | 105/4881769 | 1 |
| *Enterobacter_ludwigii* | 111/4857439 | 2 |
| *Porphyromonas_somerae* | 323/2355993 | 2 |
| *Bacillus_altitudinis* | 116/3812576 | 1 |
| *Bacillus_coagulans* | 124/3694837 | 1 |
| *Enterobacter_cloacae_complex_Hoffmann_cluster_IV* | 98/4748414 | 1 |
| *Aerococcus_viridans* | 258/2199877 | 2 |
| *Finegoldia_magna* | 256/1797577 | 2 |
| *Ketogulonicigenium_vulgare* | 202/3032608 | 2 |
| *Lautropia_mirabilis* | 245/3151995 | 2 |
| *Novosphingobium_aromaticivorans* | 197/3561584 | 2 |
| *Paracoccus_yeei* | 243/4429585 | 2 |
| *Veillonella_atypica* | 118/2099783 | 2 |
| *Actinomyces_oris* | 137/3042917 | 1 |
| *Actinomyces_radicidentis* | 103/3051613 | 1 |
| *Deinococcus_radiodurans* | 125/3060986 | 1 |
| *Exiguobacterium_AT1b* | 117/2999895 | 1 |
| *Exiguobacterium_MH3* | 127/3164195 | 1 |
| *Micrococcus_luteus* | 113/2501097 | 1 |
| *Micrococcus_lylae* | 104/2686748 | 1 |
| *Rothia_aeria* | 126/2603498 | 1 |
| *Rothia_mucilaginosa* | 90/2264603 | 1 |
| *Sphingobium_japonicum* | 112/4196714 | 1 |
| *Sphingobium_xenophagum* | 105/4487790 | 1 |
| *Treponema_denticola* | 128/2843201 | 1 |
| *Treponema_vincentii* | 110/2693513 | 1 |
| *Deinococcus_proteolyticus* | 111/2147060 | 0 |
| *Marinobacter_adhaerens* | 104/4421911 | 0 |
| *Marinobacter_hydrocarbonoclasticus* | 121/3989480 | 0 |
| *Sphingobium_SYK* | 100/4199332 | 0 |
| *Abiotrophia_defectiva* | 115/2043469 | 1 |
| *Aeromonas_caviae* | 110/4787529 | 1 |
| *Asticcacaulis_excentricus* | 126/3904170 | 1 |
| *Brachybacterium_faecium* | 101/3614992 | 1 |
| *Cardiobacterium_valvarum* | 117/2554505 | 1 |
| *Delftia_tsuruhatensis* | 106/7195716 | 1 |
| *Dermabacter_hominis* | 111/2510880 | 1 |
| *Dolosigranulum_pigrum* | 118/1862145 | 1 |
| *Flavobacterium_indicum* | 95/2993089 | 1 |
| *Gemella_morbillorum* | 135/1753088 | 1 |
| *Kallipyga_massiliensis* | 107/1770690 | 1 |
| *Kocuria_palustris* | 224/2854447 | 1 |
| *Legionella_anisa* | 114/4399396 | 1 |
| *Leuconostoc_citreum* | 105/1796284 | 1 |
| *Lysinibacillus_fusiformis* | 81/4843789 | 1 |
| *Macrococcus_caseolyticus* | 135/2102324 | 1 |
| *Methylobacterium_aquaticum* | 115/5348274 | 1 |
| *Microbacterium_oleivorans* | 104/2916866 | 1 |
| *Oceanobacillus_massiliensis* | 72/3594051 | 1 |
| *Polynucleobacter_necessarius* | 124/2159490 | 1 |
| *Psychrobacter_PRwf* | 109/2978976 | 1 |
| *Roseomonas_mucosa* | 103/4865340 | 1 |
| *Shewanella_oneidensis* | 129/4969811 | 1 |
| *Sphingopyxis_alaskensis* | 98/3345170 | 1 |
| *Thermobacillus_composti* | 127/4206343 | 1 |
| *Thermobispora_bispora* | 105/4189976 | 1 |
| *Yersinia_enterocolitica* | 111/4807490 | 1 |
| *Bradyrhizobium_BTAi1* | 53/8264687 | 0 |
| *Bradyrhizobium_S23321* | 113/7231841 | 0 |
| *Bradyrhizobium_japonicum* | 117/9207384 | 0 |
| *Paenibacillus_Y412MC10* | 111/7121665 | 0 |
| *Rhizobium_etli* | 215/4598466 | 0 |
| *Streptomyces_griseus* | 97/8545929 | 0 |
| *Tissierellia_bacterium_S7_1_4* | 100/1840142 | 1 |
| *Veillonellaceae_bacterium_DNF00626* | 117/1594238 | 1 |
| *butyrate_producing_bacterium* | 116/3601020 | 1 |
| *Alicycliphilus_denitrificans* | 131/4995263 | 0 |
| *Comamonas_kerstersii* | 108/3734555 | 0 |
| *Coriobacteriales_bacterium_DNF00809* | 124/1698563 | 0 |
| *Escherichia_coli* | 268/5399183 | 0 |
| *Ethanoligenens_harbinense* | 120/3008576 | 0 |
| *Hyphomicrobium_MC1* | 85/4757528 | 0 |
| *Janthinobacterium_Marseille* | 118/4110251 | 0 |
| *Kurthia_massiliensis* | 60/3231437 | 0 |
| *Pelagibacterium_halotolerans* | 117/3944837 | 0 |
| *Rhodospirillum_centenum* | 89/4355543 | 0 |
| *Saccharomonospora_viridis* | 115/4308349 | 0 |
| *Saccharopolyspora_erythraea* | 86/8212805 | 0 |
| *Salmonella_typhimurium* | 244/4933631 | 0 |
| *Shigella_boydii* | 121/4615997 | 0 |
| *Shigella_dysenteriae* | 88/4369232 | 0 |
| *Shigella_sonnei* | 235/4878853 | 0 |
| *Sinorhizobium_fredii* | 104/6476459 | 0 |
| *Thioalkalivibrio_sulfidophilus* | 354/3464554 | 0 |
| *Tissierellia_bacterium_S5_A11* | 107/3291852 | 0 |
| *Verminephrobacter_eiseniae* | 122/5566749 | 0 |

**Data for Case 2**

| **Species** | **Genomic Coverage** | **Aligned Reads** |
| --- | --- | --- |
| *Burkholderia_contaminans* | 146144/8509269 | 418 |
| *Burkholderia_ubonensis* | 85808/8028779 | 168 |
| *Burkholderia_multivorans* | 44193/7281887 | 141 |
| *Burkholderia_lata* | 37647/8676277 | 27 |
| *Burkholderia_stabilis* | 27001/8527967 | 24 |
| *Burkholderia_pyrrocinia* | 22399/7847882 | 20 |
| *Burkholderia_cepacia* | 22279/8396158 | 19 |
| *Burkholderia_cenocepacia* | 21226/8531679 | 18 |
| *Burkholderia_glumae* | 6008/6733840 | 15 |
| *Burkholderia_ambifaria* | 19757/7484986 | 14 |
| *Burkholderia_oklahomensis* | 12730/7313683 | 14 |
| *Burkholderia_metallica* | 19643/7424260 | 8 |
| *Burkholderia_diffusa* | 16859/6857853 | 7 |
| *Burkholderia_dolosa* | 25131/6409115 | 7 |
| *Burkholderia_latens* | 15047/6569017 | 7 |
| *Burkholderia_KJ006* | 10903/6584551 | 6 |
| *Burkholderia_gladioli* | 4793/8114449 | 5 |
| *Burkholderia_anthina* | 21503/7273081 | 3 |
| *Burkholderia_pseudomallei* | 5767/7446579 | 3 |
| *Burkholderia_thailandensis* | 8672/6739510 | 3 |
| *Burkholderia_RPE64* | 799/5379596 | 2 |
| *Burkholderia_CCGE1002* | 2310/7395722 | 1 |
| *Burkholderia_vietnamiensis* | 10927/6827896 | 1 |
| *Burkholderia_xenovorans* | 1374/9731138 | 1 |
| *Burkholderia_YI23* | 1014/6473869 | 1 |
| *Propionibacterium_acnes* | 84400/2560282 | 627 |
| *Propionibacterium_humerusii* | 1197/2644116 | 7 |
| *Acinetobacter_baumannii* | 11215/4086879 | 60 |
| *Acinetobacter_johnsonii* | 1601/3509795 | 11 |
| *Acinetobacter_junii* | 859/3782588 | 5 |
| *Acinetobacter_lwoffii* | 241/2970206 | 2 |
| *Acinetobacter_beijerinckii* | 122/3563374 | 1 |
| *Acinetobacter_calcoaceticus* | 208/3862530 | 1 |
| *Acinetobacter_gerneri* | 444/4595582 | 1 |
| *Acinetobacter_oleivorans* | 480/4152543 | 1 |
| *Acinetobacter_parvus* | 205/3606729 | 1 |
| *Acinetobacter_soli* | 158/3463526 | 1 |
| *Acinetobacter_ursingii* | 554/3825524 | 1 |
| *Staphylococcus_epidermidis* | 7196/2616530 | 51 |
| *Staphylococcus_hominis* | 2047/1140916 | 16 |
| *Staphylococcus_haemolyticus* | 1606/2685015 | 13 |
| *Staphylococcus_capitis* | 1675/2466594 | 12 |
| *Staphylococcus_cohnii* | 408/2677922 | 2 |
| *Staphylococcus_arlettae* | 101/2566235 | 1 |
| *Staphylococcus_aureus* | 261/2898306 | 1 |
| *Staphylococcus_caprae* | 373/2629820 | 1 |
| *Staphylococcus_warneri* | 843/2486042 | 1 |
| *Gordonia_sputi* | 10534/4954549 | 87 |
| *Gordonia_polyisoprenivorans* | 849/5669805 | 7 |
| *Corynebacterium_accolens* | 2078/2465976 | 19 |
| *Corynebacterium_durum* | 564/2809766 | 5 |
| *Corynebacterium_matruchotii* | 417/2992345 | 3 |
| *Corynebacterium_pseudogenitalium* | 2896/2601506 | 3 |
| *Corynebacterium_afermentans* | 324/2345845 | 2 |
| *Corynebacterium_amycolatum* | 238/2514382 | 2 |
| *Corynebacterium_jeikeium* | 283/2462499 | 2 |
| *Corynebacterium_tuberculostearicum* | 2683/2372621 | 2 |
| *Corynebacterium_ureicelerivorans* | 364/2279990 | 2 |
| *Corynebacterium_aurimucosum* | 136/2790189 | 1 |
| *Corynebacterium_coyleae* | 69/2568946 | 1 |
| *Corynebacterium_imitans* | 127/2565321 | 1 |
| *Corynebacterium_kroppenstedtii* | 126/2446804 | 1 |
| *Corynebacterium_lipophiloflavum* | 110/2386544 | 1 |
| *Corynebacterium_propinquum* | 258/2553780 | 1 |
| *Corynebacterium_pseudodiphtheriticum* | 128/2328531 | 1 |
| *Corynebacterium_simulans* | 278/2737971 | 1 |
| *Brevundimonas_vesicularis* | 7012/3358839 | 57 |
| *Brevundimonas_subvibrioides* | 807/3445263 | 3 |
| *Brevundimonas_diminuta* | 249/3369386 | 2 |
| *Methylobacterium_radiotolerans* | 3640/6077833 | 16 |
| *Methylobacterium_brachiatum* | 590/5807713 | 2 |
| *Methylobacterium_mesophilicum* | 651/6214729 | 2 |
| *Methylobacterium_aquaticum* | 197/5348274 | 1 |
| *Methylobacterium_chloromethanicum* | 681/5777908 | 1 |
| *Methylobacterium_extorquens* | 463/5943768 | 1 |
| *Methylobacterium_populi* | 235/5800441 | 1 |
| *Cupriavidus_metallidurans* | 4651/3928089 | 31 |
| *Cupriavidus_gilardii* | 1601/5578753 | 12 |
| *Cupriavidus_nantongensis* | 816/7075719 | 2 |
| *Cupriavidus_necator* | 485/6557542 | 2 |
| *Cupriavidus_pauculus* | 603/6829059 | 1 |
| *Cupriavidus_taiwanensis* | 311/5919322 | 1 |
| *Sphingomonas_parapaucimobilis* | 2672/3995782 | 21 |
| *Sphingomonas_melonis* | 1576/4156476 | 11 |
| *Sphingomonas_adhaesiva* | 1092/4026130 | 6 |
| *Sphingomonas_echinoides* | 1190/4264986 | 4 |
| *Sphingomonas_MM* | 473/4054833 | 3 |
| *Moraxella_osloensis* | 18485/2434688 | 41 |
| *Moraxella_atlantae* | 480/2289665 | 4 |
| *Enhydrobacter_aerosaccus* | 17851/2856138 | 42 |
| *Acidovorax_KKS102* | 4335/5196935 | 32 |
| *Acidovorax_avenae* | 385/5482170 | 1 |
| *Acidovorax_citrulli* | 753/5352772 | 1 |
| *Acidovorax_ebreus* | 764/3796573 | 1 |
| *Acidovorax_JS42* | 1017/4448856 | 1 |
| *Streptococcus_gordonii* | 1277/2196662 | 9 |
| *Streptococcus_oralis* | 558/1958690 | 3 |
| *Streptococcus_sanguinis* | 779/2388435 | 4 |
| *Streptococcus_cristatus* | 617/2142100 | 2 |
| *Streptococcus_sinensis* | 240/2062993 | 2 |
| *Streptococcus_anginosus* | 330/2233640 | 1 |
| *Streptococcus_pseudopneumoniae* | 246/2190731 | 1 |
| *Micrococcus_luteus* | 3044/2501097 | 24 |
| *Micrococcus_terreus* | 756/3087820 | 5 |
| *Enterobacter_cloacae_complex_Hoffmann_cluster_IV* | 2249/4748414 | 10 |
| *Enterobacter_asburiae* | 252/4753402 | 1 |
| *Rothia_dentocariosa* | 1904/2506025 | 15 |
| *Rothia_aeria* | 1338/2603498 | 10 |
| *Rothia_mucilaginosa* | 107/2264603 | 1 |
| *Cutibacterium_granulosum* | 1786/2141325 | 15 |
| *Propionibacterium_namnetense* | 1895/2369944 | 7 |
| *Cutibacterium_avidum* | 972/2729848 | 3 |
| *Methylotenera_301* | 2270/3059871 | 21 |
| *Ralstonia_insidiosa* | 2259/5808308 | 17 |
| *Ralstonia_pickettii* | 105/8125850 | 1 |
| *Kocuria_polaris* | 1609/3834128 | 13 |
| *Kocuria_kristinae* | 263/2409808 | 2 |
| *Kocuria_palustris* | 412/2854447 | 2 |
| *Kocuria_rhizophila* | 237/2697540 | 2 |
| *Kocuria_varians* | 154/2901386 | 1 |
| *Prevotella_melaninogenica* | 1159/3168282 | 6 |
| *Prevotella_intermedia* | 220/2699437 | 2 |
| *Prevotella_nigrescens* | 271/2987072 | 2 |
| *Prevotella_baroniae* | 87/3129177 | 1 |
| *Prevotella_oris* | 125/3346788 | 1 |
| *Actinomyces_israelii* | 95/4026782 | 1 |
| *Actinomyces_odontolyticus* | 378/2432045 | 1 |
| *Actinomyces_timonensis* | 48/2932974 | 1 |
| *Actinomyces_viscosus* | 811/3134536 | 1 |
| *Pseudomonas_stutzeri* | 1324/4650155 | 10 |
| *Pseudomonas_mendocina* | 218/5434353 | 2 |
| *Pseudomonas_fulva* | 126/4920769 | 1 |
| *Pseudomonas_monteilii* | 76/6000087 | 1 |
| *Pseudomonas_oryzihabitans* | 124/4834356 | 1 |
| *Microbacterium_aurum* | 418/3424892 | 3 |
| *Microbacterium_laevaniformans* | 190/3000545 | 2 |
| *Microbacterium_hominis* | 333/3506963 | 1 |
| *Microbacterium_resistens* | 90/3983164 | 1 |
| *Microbacterium_testaceum* | 484/3982034 | 1 |
| *Haemophilus_parainfluenzae* | 1429/2086875 | 11 |
| *Sphingobium_xenophagum* | 916/4487790 | 6 |
| *Mesorhizobium_loti* | 510/7036071 | 3 |
| *Sphingobium_yanoikuyae* | 847/5532659 | 3 |
| *Haemophilus_parahaemolyticus* | 127/2038028 | 1 |
| *Lactobacillus_crispatus* | 468/2043161 | 4 |
| *Lactobacillus_iners* | 357/1277759 | 3 |
| *Lactobacillus_curvatus* | 322/1986991 | 1 |
| *Lactobacillus_amylovorus* | 114/2067702 | 0 |
| *Dolosigranulum_pigrum* | 1146/1862145 | 9 |
| *Polynucleobacter_necessarius* | 1144/2159490 | 10 |
| *Mycobacterium_iranicum* | 387/6138463 | 3 |
| *Bradyrhizobium_japonicum* | 459/9207384 | 1 |
| *Bradyrhizobium_ORS* | 830/7456587 | 1 |
| *Bradyrhizobium_S23321* | 341/7231841 | 1 |
| *Clostridium_haemolyticum* | 508/2882215 | 4 |
| *Clostridium_leptum* | 267/3270409 | 3 |
| *Paracoccus_yeei* | 580/4429585 | 4 |
| *Flavobacterium_branchiophilum* | 386/3559884 | 2 |
| *Flavobacterium_indicum* | 135/2993089 | 1 |
| *Flavobacterium_johnsoniae* | 364/6096872 | 1 |
| *Porphyromonas_somerae* | 590/2355993 | 5 |
| *Rhodococcus_fascians* | 499/5139988 | 4 |
| *Rhodococcus_erythropolis* | 297/6516310 | 2 |
| *Roseomonas_cervicalis* | 269/5105947 | 2 |
| *Roseomonas_mucosa* | 440/4865340 | 2 |
| *Neisseria_mucosa* | 112/2169497 | 1 |
| *Neisseria_perflava* | 638/3786149 | 1 |
| *Porphyromonas_catoniae* | 94/2101655 | 1 |
| *Escherichia_vulneris* | 602/4375121 | 5 |
| *Rhodoferax_ferrireducens* | 781/4712337 | 5 |
| *Psychrobacter_PRwf* | 522/2978976 | 2 |
| *Delftia_tsuruhatensis* | 794/7195716 | 1 |
| *Alcaligenes_faecalis* | 356/4233756 | 3 |
| *Azospira_oryzae* | 562/3806980 | 3 |
| *Cellvibrio_japonicus* | 799/4576573 | 4 |
| *Finegoldia_magna* | 344/1797577 | 3 |
| *Lawsonella_clevelandensis* | 306/1915154 | 3 |
| *Novosphingobium_aromaticivorans* | 465/3561584 | 3 |
| *Capnocytophaga_granulosa* | 240/2746468 | 2 |
| *Gemella_morbillorum* | 293/1753088 | 2 |
| *Xanthomonas_campestris* | 212/4908383 | 2 |
| *Bacillus_megaterium* | 190/5343114 | 1 |
| *Capnocytophaga_gingivalis* | 129/2667498 | 1 |
| *Capnocytophaga_sputigena* | 141/2998485 | 1 |
| *Chryseobacterium_gleum* | 425/5570680 | 1 |
| *Chryseobacterium_indologenes* | 294/5832495 | 1 |
| *Gemella_haemolysans* | 143/2053699 | 1 |
| *Novosphingobium_PP1Y* | 113/3911486 | 1 |
| *Veillonella_parvula* | 331/2132142 | 1 |
| *Achromobacter_xylosoxidans* | 201/6813182 | 2 |
| *Aggregatibacter_segnis* | 284/2012050 | 2 |
| *Asticcacaulis_excentricus* | 272/3904170 | 2 |
| *Cloacibacterium_normanense* | 211/2736686 | 2 |
| *Comamonas_testosteroni* | 330/5373644 | 2 |
| *Granulicatella_adiacens* | 206/1947256 | 2 |
| *Haematobacter_missouriensis* | 394/3960158 | 2 |
| *Herminiimonas_arsenicoxydans* | 831/3424307 | 2 |
| *Massilia_timonae* | 327/6136630 | 2 |
| *Pelagibacterium_halotolerans* | 291/3944837 | 2 |
| *Ramlibacter_tataouinensis* | 296/4070193 | 2 |
| *Sinorhizobium_meliloti* | 233/6813723 | 2 |
| *Sphingopyxis_alaskensis* | 426/3345170 | 2 |
| *Arthrobacter_arilaitensis* | 102/3859257 | 1 |
| *Arthrobacter_phenanthrenivorans* | 131/4250414 | 1 |
| *Hyphomicrobium_denitrificans* | 83/3808687 | 1 |
| *Klebsiella_aerogenes* | 72/5452368 | 1 |
| *Klebsiella_pneumoniae* | 132/5574202 | 1 |
| *Pseudoxanthomonas_spadix* | 134/3452554 | 1 |
| *Pseudoxanthomonas_suwonensis* | 217/3419049 | 1 |
| *Rhizobium_etli* | 122/4598466 | 1 |
| *Rhizobium_tropici* | 96/3837060 | 1 |
| *Acholeplasma_brassicae* | 130/1877792 | 1 |
| *Actinomadura_madurae* | 140/10255684 | 1 |
| *Aerococcus_viridans* | 146/2199877 | 1 |
| *Aeromonas_caviae* | 114/4787529 | 1 |
| *Alicycliphilus_denitrificans* | 426/4995263 | 1 |
| *Alistipes_putredinis* | 100/2550788 | 1 |
| *Atopobium_rimae* | 99/1630016 | 1 |
| *Blastococcus_saxobsidens* | 97/4875340 | 1 |
| *Brevibacterium_linens* | 113/4209935 | 1 |
| *Budvicia_aquatica* | 136/5669921 | 1 |
| *Campylobacter_concisus* | 135/2052007 | 1 |
| *Cellulomonas_flavigena* | 120/4123179 | 1 |
| *Curtobacterium_pusillum* | 135/3973750 | 1 |
| *Deinococcus_geothermalis* | 163/2467205 | 1 |
| *Dermabacter_vaginalis* | 90/2392314 | 1 |
| *Dialister_invisus* | 146/1895960 | 1 |
| *Empedobacter_falsenii* | 112/3715858 | 1 |
| *Erythrobacter_litoralis* | 379/3052398 | 1 |
| *Ewingella_americana* | 182/4868720 | 1 |
| *Exiguobacterium_AT1b* | 115/2999895 | 1 |
| *Geobacter_lovleyi* | 129/3917761 | 1 |
| *Kingella_oralis* | 105/2406715 | 1 |
| *Kytococcus_sedentarius* | 131/2785024 | 1 |
| *Leptospira_noguchii* | 145/4763760 | 1 |
| *Methylophaga_JAM7* | 239/2697465 | 1 |
| *Modestobacter_marinus* | 238/5575517 | 1 |
| *Nitrosomonas_eutropha* | 80/2661057 | 1 |
| *Nocardioides_JS614* | 329/4985871 | 1 |
| *Oribacterium_sinus* | 157/2707374 | 1 |
| *Paludibacter_propionicigenes* | 84/3685504 | 1 |
| *Pandoraea_norimbergensis* | 278/6167370 | 1 |
| *Pantoea_vagans* | 108/4024986 | 1 |
| *Peptostreptococcus_stomatis* | 133/1988774 | 1 |
| *Phenylobacterium_zucineum* | 204/3996255 | 1 |
| *Polaromonas_JS666* | 246/5200264 | 1 |
| *Rhodobacter_sphaeroides* | 134/4450439 | 1 |
| *Ruminococcus_torques* | 84/3341681 | 1 |
| *Tannerella_forsythia* | 128/3405521 | 1 |
| *Tatumella_ptyseos* | 110/3533287 | 1 |
| *Treponema_vincentii* | 126/2693513 | 1 |
| *Variovorax_paradoxus* | 293/7148516 | 1 |
| *Vibrio_EJY3* | 100/5452646 | 1 |
| *Tissierellia_bacterium_S5_A11* | 159/3291852 | 1 |

**Data for Case 3**

| **Species** | **Genomic Coverage** | **Aligned Reads** | |
| --- | --- | --- | --- |
| *Propionibacterium_acnes* | 105291/2560282 | 747 |  |
| *Propionibacterium_humerusii* | 1589/2644116 | 3 |  |
| *Ralstonia_insidiosa* | 23013/5808308 | 152 |  |
| *Ralstonia_mannitolilytica* | 1351/4881769 | 6 |  |
| *Ralstonia_solanacearum* | 350/3984240 | 2 |  |
| *Bradyrhizobium_japonicum* | 16455/9207384 | 39 |  |
| *Bradyrhizobium_S23321* | 13537/7231841 | 33 |  |
| *Bradyrhizobium_BTAi1* | 1468/8264687 | 7 |  |
| *Brevundimonas_vesicularis* | 8261/3358839 | 64 |  |
| *Brevundimonas_subvibrioides* | 272/3445263 | 2 |  |
| *Corynebacterium_accolens* | 2702/2465976 | 20 |  |
| *Corynebacterium_matruchotii* | 1753/2992345 | 13 |  |
| *Corynebacterium_singulare* | 484/2830519 | 4 |  |
| *Corynebacterium_striatum* | 436/2915083 | 4 |  |
| *Corynebacterium_tuberculostearicum* | 552/2372621 | 2 |  |
| *Corynebacterium_afermentans* | 132/2345845 | 1 |  |
| *Corynebacterium_callunae* | 123/2839551 | 1 |  |
| *Corynebacterium_diphtheriae* | 204/2535346 | 1 |  |
| *Corynebacterium_kroppenstedtii* | 131/2446804 | 1 |  |
| *Corynebacterium_pilosum* | 123/2531972 | 1 |  |
| *Corynebacterium_resistens* | 111/2601311 | 1 |  |
| *Corynebacterium_simulans* | 267/2737971 | 1 |  |
| *Cutibacterium_granulosum* | 3312/2141325 | 22 |  |
| *Sphingomonas_melonis* | 2754/4156476 | 20 |  |
| *Sphingomonas_echinoides* | 1568/4264986 | 13 |  |
| *Propionibacterium_namnetense* | 2306/2369944 | 7 |  |
| *Sphingomonas_adhaesiva* | 699/4026130 | 4 |  |
| *Cutibacterium_avidum* | 809/2729848 | 2 |  |
| *Acinetobacter_johnsonii* | 3341/3509795 | 27 |  |
| *Acinetobacter_lwoffii* | 286/2970206 | 2 |  |
| *Acinetobacter_schindleri* | 103/3415006 | 1 |  |
| *Methylobacterium_radiotolerans* | 959/6077833 | 6 |  |
| *Methylobacterium_mesophilicum* | 1618/6214729 | 4 |  |
| *Methylobacterium_chloromethanicum* | 412/5777908 | 2 |  |
| *Methylobacterium_populi* | 683/5800441 | 2 |  |
| *Staphylococcus_epidermidis* | 1557/2616530 | 12 |  |
| *Staphylococcus_hominis* | 355/1140916 | 3 |  |
| *Staphylococcus_capitis* | 143/2466594 | 1 |  |
| *Staphylococcus_gallinarum* | 143/3174430 | 1 |  |
| *Staphylococcus_haemolyticus* | 136/2685015 | 1 |  |
| *Staphylococcus_pettenkoferi* | 116/2502346 | 1 |  |
| *Staphylococcus_sciuri* | 132/2744306 | 1 |  |
| *Staphylococcus_warneri* | 155/2486042 | 1 |  |
| *Pseudomonas_stutzeri* | 1387/4650155 | 11 |  |
| *Pseudomonas_mendocina* | 260/5434353 | 2 |  |
| *Pseudomonas_fluorescens* | 124/6136735 | 1 |  |
| *Actinomyces_johnsonii* | 1052/3386293 | 5 |  |
| *Actinomyces_massiliensis* | 138/3371154 | 1 |  |
| *Actinomyces_viscosus* | 386/3134536 | 1 |  |
| *Micrococcus_luteus* | 827/2501097 | 7 |  |
| *Micrococcus_terreus* | 278/3087820 | 2 |  |
| *Enhydrobacter_aerosaccus* | 5083/2856138 | 8 |  |
| *Neisseria_perflava* | 444/3786149 | 2 |  |
| *Neisseria_cinerea* | 140/1873113 | 1 |  |
| *Streptococcus_gordonii* | 275/2196662 | 1 |  |
| *Streptococcus_mitis* | 75/2146611 | 1 |  |
| *Moraxella_osloensis* | 5321/2434688 | 7 |  |
| *Delftia_tsuruhatensis* | 919/7195716 | 5 |  |
| *Mesorhizobium_ciceri* | 782/6264489 | 1 |  |
| *Mesorhizobium_loti* | 241/7036071 | 1 |  |
| *Mesorhizobium_opportunistum* | 239/6884444 | 1 |  |
| *Rhodococcus_fascians* | 620/5139988 | 5 |  |
| *Kocuria_palustris* | 310/2854447 | 3 |  |
| *Rothia_dentocariosa* | 384/2506025 | 3 |  |
| *Kocuria_polaris* | 122/3834128 | 1 |  |
| *Kocuria_rhizophila* | 68/2697540 | 1 |  |
| *Prevotella_bivia* | 134/2580302 | 1 |  |
| *Prevotella_buccalis* | 139/3035131 | 1 |  |
| *Prevotella_intermedia* | 142/2699437 | 1 |  |
| *Prevotella_melaninogenica* | 285/3168282 | 1 |  |
| *Rothia_aeria* | 153/2603498 | 1 |  |
| *Rothia_mucilaginosa* | 123/2264603 | 1 |  |
| *Bacillus_smithii* | 549/3368778 | 4 |  |
| *Paracoccus_versutus* | 365/5502608 | 1 |  |
| *Paracoccus_yeei* | 235/4429585 | 1 |  |
| *Campylobacter_mucosalis* | 364/1752144 | 2 |  |
| *Gemella_sanguinis* | 244/1795754 | 2 |  |
| *Porphyromonas_somerae* | 248/2355993 | 2 |  |
| *Campylobacter_gracilis* | 108/2281652 | 1 |  |
| *Gemella_haemolysans* | 130/2053699 | 1 |  |
| *Porphyromonas_gingivalis* | 143/2378872 | 1 |  |
| *Deinococcus_geothermalis* | 274/2467205 | 2 |  |
| *Dolosigranulum_pigrum* | 267/1862145 | 2 |  |
| *Lactobacillus_iners* | 142/1277759 | 2 |  |
| *Rubrobacter_xylanophilus* | 296/3225748 | 2 |  |
| *Thauera_MZ1T* | 154/4496212 | 2 |  |
| *Tsukamurella_tyrosinosolvens* | 288/5281148 | 2 |  |
| *Dietzia_maris* | 190/3505932 | 1 |  |
| *Haemophilus_parainfluenzae* | 149/2086875 | 1 |  |
| *Haemophilus_sputorum* | 118/2144403 | 1 |  |
| *Ochrobactrum_anthropi* | 655/4783208 | 1 |  |
| *Ochrobactrum_intermedium* | 895/4665240 | 1 |  |
| *Roseomonas_gilardii* | 127/5030491 | 1 |  |
| *Sinorhizobium_meliloti* | 139/6813723 | 1 |  |
| *Aerococcus_viridans* | 152/2199877 | 1 |  |
| *Aggregatibacter_segnis* | 152/2012050 | 1 |  |
| *Alcanivorax_borkumensis* | 89/3120143 | 1 |  |
| *Anoxybacillus_flavithermus* | 104/2846746 | 1 |  |
| *Brevibacterium_casei* | 144/3769980 | 1 |  |
| *Candidatus_Accumulibacter_phosphatis* | 100/5058518 | 1 |  |
| *Clostridium_leptum* | 141/3270409 | 1 |  |
| *Comamonas_aquatica* | 137/3764434 | 1 |  |
| *Enterobacter_cloacae_complex_Hoffmann_cluster_IV* | 123/4748414 | 1 |  |
| *Enterococcus_cecorum* | 124/2438959 | 1 |  |
| *Eubacterium_rectale* | 121/3698419 | 1 |  |
| *Finegoldia_magna* | 123/1797577 | 1 |  |
| *Herminiimonas_arsenicoxydans* | 110/3424307 | 1 |  |
| *Lawsonella_clevelandensis* | 128/1915154 | 1 |  |
| *Leclercia_adecarboxylata* | 242/4803917 | 1 |  |
| *Massilia_timonae* | 127/6136630 | 1 |  |
| *Nitrobacter_winogradskyi* | 106/3402093 | 1 |  |
| *Oscillatoria_PCC* | 153/7479014 | 1 |  |
| *Pseudoglutamicibacter_albus* | 126/2476958 | 1 |  |
| *Pseudoxanthomonas_Mexicana* | 124/3943279 | 1 |  |
| *Psychrobacter_PRwf* | 142/2978976 | 1 |  |
| *Rhodopseudomonas_palustris* | 349/5744041 | 1 |  |
| *Stenotrophomonas_maltophilia* | 129/4509724 | 1 |  |
| *Streptomyces_coelicolor* | 151/8667507 | 1 |  |
| *Tannerella_forsythia* | 134/3405521 | 1 |  |
| *Thermoanaerobacterium_thermosaccharolyticum* | 118/2785752 | 1 |  |
| *Veillonella_parvula* | 127/2132142 | 1 |  |
| *Xanthomonas_campestris* | 133/4908383 | 1 |  |

**Data for Case 4**

| **Species** | **Genomic Coverage** | **Aligned Reads** |  |
| --- | --- | --- | --- |
| *Propionibacterium_acnes* | 23603/2560282 | 164 | |
| *Bradyrhizobium_BTAi1* | 2125/8264687 | 9 | |
| *Bradyrhizobium_japonicum* | 6017/9207384 | 7 | |
| *Bradyrhizobium_S23321* | 4781/7231841 | 6 | |
| *Bradyrhizobium_oligotrophicum* | 1308/8264165 | 3 | |
| *Corynebacterium_pseudogenitalium* | 1400/2601506 | 4 | |
| *Corynebacterium_accolens* | 635/2465976 | 3 | |
| *Corynebacterium_striatum* | 327/2915083 | 3 | |
| *Corynebacterium_tuberculostearicum* | 1035/2372621 | 3 | |
| *Corynebacterium_afermentans* | 261/2345845 | 2 | |
| *Corynebacterium_aurimucosum* | 348/2790189 | 2 | |
| *Corynebacterium_lipophiloflavum* | 252/2386544 | 2 | |
| *Corynebacterium_singulare* | 340/2830519 | 2 | |
| *Corynebacterium_jeikeium* | 123/2462499 | 1 | |
| *Corynebacterium_matruchotii* | 141/2992345 | 1 | |
| *Corynebacterium_pseudodiphtheriticum* | 109/2328531 | 1 | |
| *Ralstonia_insidiosa* | 2552/5808308 | 12 | |
| *Ralstonia_mannitolilytica* | 396/4881769 | 1 | |
| *Acinetobacter_johnsonii* | 850/3509795 | 6 | |
| *Acinetobacter_baumannii* | 273/4086879 | 1 | |
| *Acinetobacter_guillouiae* | 129/4905802 | 1 | |
| *Acinetobacter_haemolyticus* | 132/3715798 | 1 | |
| *Acinetobacter_junii* | 69/3782588 | 1 | |
| *Methylobacterium_radiotolerans* | 1186/6077833 | 8 | |
| *Methylobacterium_populi* | 118/5800441 | 1 | |
| *Staphylococcus_epidermidis* | 712/2616530 | 5 | |
| *Staphylococcus_capitis* | 267/2466594 | 2 | |
| *Staphylococcus_cohnii* | 134/2677922 | 1 | |
| *Staphylococcus_hominis* | 122/1140916 | 1 | |
| *Cutibacterium_granulosum* | 1030/2141325 | 8 | |
| *Propionibacterium_namnetense* | 194/2369944 | 1 | |
| *Sphingomonas_melonis* | 780/4156476 | 7 | |
| *Sphingomonas_parapaucimobilis* | 117/3995782 | 1 | |
| *Brevundimonas_vesicularis* | 844/3358839 | 7 | |
| *Enhydrobacter_aerosaccus* | 1606/2856138 | 5 | |
| *Marinobacter_adhaerens* | 226/4421911 | 1 | |
| *Streptococcus_pseudopneumoniae* | 171/2190731 | 1 | |
| *Streptococcus_sanguinis* | 132/2388435 | 1 | |
| *Klebsiella_oxytoca* | 418/6229565 | 3 | |
| *Serinicoccus_chungangensis* | 396/3557308 | 3 | |
| *Micrococcus_luteus* | 478/2501097 | 1 | |
| *Pseudomonas_putida* | 139/6377271 | 1 | |
| *Abiotrophia_defectiva* | 283/2043469 | 2 | |
| *Moraxella_osloensis* | 764/2434688 | 2 | |
| *Pantoea_dispersa* | 262/4951455 | 2 | |
| *Paracoccus_yeei* | 363/4429585 | 2 | |
| *Lactobacillus_helveticus* | 115/2058319 | 1 | |
| *Peptoniphilus_duerdenii* | 140/2124994 | 1 | |
| *Peptoniphilus_lacrimalis* | 179/1849403 | 1 | |
| *Prevotella_denticola* | 130/2937589 | 1 | |
| *Prevotella_oris* | 126/3346788 | 1 | |
| *Rothia_dentocariosa* | 139/2506025 | 1 | |
| *Rothia_mucilaginosa* | 123/2264603 | 1 | |
| *Veillonella_atypica* | 288/2099783 | 1 | |
| *Actinomyces_odontolyticus* | 126/2432045 | 1 | |
| *Aeromonas_caviae* | 136/4787529 | 1 | |
| *Afipia_felis* | 146/4203390 | 1 | |
| *Aggregatibacter_segnis* | 98/2012050 | 1 | |
| *Agrobacterium_tumefaciens* | 315/5481605 | 1 | |
| *Anoxybacillus_flavithermus* | 155/2846746 | 1 | |
| *Brevibacterium_mcbrellneri* | 145/2562754 | 1 | |
| *Campylobacter_concisus* | 136/2052007 | 1 | |
| *Capnocytophaga_gingivalis* | 129/2667498 | 1 | |
| *Clostridium_haemolyticum* | 147/2882215 | 1 | |
| *Dysgonomonas_capnocytophagoides* | 105/4378358 | 1 | |
| *Gordonia_terrae* | 107/5701501 | 1 | |
| *Haemophilus_parainfluenzae* | 93/2086875 | 1 | |
| *Kocuria_palustris* | 62/2854447 | 1 | |
| *Kytococcus_sedentarius* | 125/2785024 | 1 | |
| *Lautropia_mirabilis* | 111/3151995 | 1 | |
| *Lawsonella_clevelandensis* | 123/1915154 | 1 | |
| *Massilia_timonae* | 122/6136630 | 1 | |
| *Neisseria_macacae* | 70/2749018 | 1 | |
| *Nocardioides_JS614* | 110/4985871 | 1 | |
| *Parabacteroides_distasonis* | 155/4811379 | 1 | |
| *Psychrobacter_phenylpyruvicus* | 133/3099103 | 1 | |
| *Rhodopseudomonas_palustris* | 212/5744041 | 1 | |
| *Shewanella_baltica* | 112/5347283 | 1 | |
| *Shigella_flexneri* | 114/4698633 | 1 | |
| *Solobacterium_moorei* | 131/2337626 | 1 | |
| *Xanthomonas_campestris* | 127/4908383 | 1 | |
| *Tissierellia_bacterium_S7_1_4* | 132/1840142 | 1 | |
