# Supplementary material for: Next-Generation Sequencing of Cerebrospinal Fluid for the Diagnosis of Neurocysticercosis
Source: Front Neurol. 2018 Jun 19;9:471. doi: 10.3389/fneur.2018.00471 (PMC6018529; doi:10.3389/fneur.2018.00471)
Supplement: Supplementary Table 3 — Number of reads aligning to fungal sequences. [file Table_3.DOC]

**Supplementary Table 3. Number of reads aligning to fungal sequences.**

**Data for** Case 1

| **Species** | **Genomic Coverage** | **Aligned Reads** |  |
| --- | --- | --- | --- |
| *Alternaria_alternata* | 1564/33021769 | 15 | |
| *Malassezia_globosa* | 1147/8872979 | 10 | |
| *Sordaria_macrospora* | 1108/40002837 | 3 | |
| *Candida_parapsilosis* | 245/13078718 | 2 | |
| *Debaryomyces_hansenii* | 139/12182018 | 1 | |

**Data for Case 2**

| **Species** | **Genomic Coverage** | **Aligned Reads** |  |
| --- | --- | --- | --- |
| *Malassezia_globosa* | 1119/8872979 | 9 | |
| *Sordaria_macrospora* | 2191/40002837 | 4 | |
| *Candida_parapsilosis* | 186/13078718 | 2 | |
| *Albugo_laibachii* | 590/32805071 | 1 | |
| *Alternaria_alternata* | 126/33021769 | 1 | |
| *Aspergillus_sydowii* | 134/34381986 | 1 | |
| *Debaryomyces_hansenii* | 66/12182018 | 1 | |
| *Fusarium_graminearum* | 137/36358997 | 1 | |
| *Meyerozyma_guilliermondii* | 112/10610034 | 1 | |
| *Phanerochaete_chrysosporium* | 188/29855776 | 1 | |

**Data for** Case 3

| **Species** | **Genomic Coverage** | **Aligned Reads** |  |
| --- | --- | --- | --- |
| *Malassezia_globosa* | 374/8872979 | 4 | |
| *Candida_parapsilosis* | 226/13078718 | 2 | |
| *Phanerochaete_chrysosporium* | 220/29855776 | 2 | |
| *Alternaria_alternata* | 133/33021769 | 1 | |
| *Aspergillus_sydowii* | 235/34381986 | 1 | |
| *Cyberlindnera_jadinii* | 60/13991877 | 1 | |
| *Talaromyces_marneffei* | 121/28648375 | 1 | |

**Data for** Case 4

| **Species** | **Genomic Coverage** | **Aligned Reads** |  |
| --- | --- | --- | --- |
| *Malassezia_globosa* | 115/8872979 | 1 | |
