# Supplementary material for: Next-Generation Sequencing of Cerebrospinal Fluid for the Diagnosis of Neurocysticercosis
Source: Front Neurol. 2018 Jun 19;9:471. doi: 10.3389/fneur.2018.00471 (PMC6018529; doi:10.3389/fneur.2018.00471)
Supplement: Supplementary Table 4 — Number of reads aligning to parasitic sequences. [file Table_4.DOC]

**Supplementary Table 4. Number of reads aligning to parasitic sequences.**

**Data for** Case 1

| **Species** | **Genomic Coverage** | **Aligned Reads** |
| --- | --- | --- |
| *Taenia_solium* | 817858/116854196 | 6513 |
| *Taenia_saginata* | 26761/152219323 | 41 |
| *Taenia_asiatica* | 26303/151825899 | 34 |
| *Entamoeba_dispar* | 349/27672952 | 1 |

**Data for Case 2**

| **Species** | **Genomic Coverage** | **Aligned Reads** | |
| --- | --- | --- | --- |
| *Taenia_solium* | 1339875/116854196 | | 9491 |
| *Taenia_saginata* | 39709/152219323 | | 71 |
| *Taenia_asiatica* | 33596/151825899 | | 59 |
| *Acanthamoeba_mauritaniensis* | 1110/96417684 | | 3 |
| *Necator_americanus* | 318/230537098 | | 3 |
| *Plasmodium_malariae* | 269/28786109 | | 1 |
| *Wuchereria_bancrofti* | 140/77159569 | | 1 |

**Data for Case 3**

| **Species** | **Genomic Coverage** | **Aligned Reads** |
| --- | --- | --- |
| *Taenia_solium* | 65875/116854196 | 478 |
| *Taenia_asiatica* | 1234/151825899 | 5 |
| *Trypanosoma_cruzi* | 236/81175611 | 2 |
| *Taenia_saginata* | 1660/152219323 | 1 |
| *Wuchereria_bancrofti* | 183/77159569 | 1 |

**Data for** Case 4

| **Species** | **Genomic Coverage** | **Aligned Reads** |
| --- | --- | --- |
| *Taenia_solium* | 254429/116854196 | 1784 |
| *Taenia_saginata* | 7809/152219323 | 13 |
| *Taenia_asiatica* | 6322/151825899 | 10 |
| *Enterobius_vermicularissta* | 77/111762754 | 1 |
