# Supplementary material for: Next-Generation Sequencing of Cerebrospinal Fluid for the Diagnosis of Neurocysticercosis
Source: Front Neurol. 2018 Jun 19;9:471. doi: 10.3389/fneur.2018.00471 (PMC6018529; doi:10.3389/fneur.2018.00471)
Supplement: Supplementary Table 5 — Case Definitions and Exclusion Criteria for Encephalitis and Meningitis in the Multicenter Study. [file Table_5.DOC]

**Supplementary Table 5. Case Definitions and Exclusion Criteria for Encephalitis and Meningitis in the Multicenter Study**

| **Case Definitions** **for Encephalitis in** **the Multicenter Study** |
| --- |
| **Major Criteria (required):** |
| Patients presenting to medical attention with altered mental status (defined as decreased or altered level of consciousness, lethargy or personality change) lasting ≥24 h with no alternative cause identified, and/or |
| Generalized or partial seizures not fully attributable to a preexisting seizure disorder or a simple febrile seizure. |
| **Minor Criteria (≥ 2 required):** |
| Documented fever ≥ 38℃ within the 72 h before or after presentation |
| New onset of focal neurologic findings |
| CSF WBC count ≥ 5/cubic mm |
| Abnormality of brain parenchyma on neuroimaging suggestive of encephalitis that is either new from prior studies or appears acute in onset |
| Abnormality on EEG that is consistent with encephalitis and not attributable to another cause |
| **Exclusion Criteria for Encephalitis in** **the Multicenter Study** |
| ≤28 days of age |
| Non-infectious encephalitis, such as autoimmune disorders, paraneoplastic syndromes, NMOSD, neuropsychiatric involvement of rheumatic diseases (systemic lupus erythematosus, Bechet's syndrome, systemic vasculitis, et al.) |
| HIV or syphilis infection |
| A history of recent (within 4 weeks before the onset of disease) vaccination |
| Meningitis without clinical brain parenchyma involvement |
| Absolute contraindications for lumbar puncture |
| Traumatic LP with obvious blood-contaminated CSF |
| Pregnancy |
| Without informed consent |
| **Case Definitions for Meningitis in** **the Multicenter Study** |
| Patients presenting to medical attention with at least two of the four symptoms of: headache, fever (documented fever ≥ 38℃ within the 72 h before or after presentation), neck stiffness, decreased level of consciousness (defined by a Glasgow Coma Scale score below 14); and |
| Confirmed by lumber puncture (CSF WBC count ≥ 5/cubic mm). |
| **Exclusion Criteria for Meningitis in the Multicenter Study** |
| ≤28 days of age |
| HIV or syphilis infection |
| Meningeal malignancy confirmed by CSF cytology |
| Traumatic LP with obvious blood-contaminated CSF |
| Pregnancy |
| Without informed consent |
